# Fecal Streptococcus Alteration Is Associated with Gastric Cancer Occurrence and Liver Metastasis

**DOI:** 10.1128/mBio.02994-21

**Published:** 2021-12-07

**Authors:** Dandan Yu, Jinru Yang, Min Jin, Bin Zhou, Linli Shi, Lei Zhao, Jieying Zhang, Zhenyu Lin, Jinghua Ren, Li Liu, Tao Zhang, Hongli Liu

**Affiliations:** a Cancer Center, Union Hospital, Tongji Medical College, Huazhong University of Science and Technology, Wuhan, People’s Republic of China; b Department of Epidemiology and Biostatistics, the Ministry of Education Key Lab of Environment and Health, School of Public Health, Huazhong University of Science and Technology, Wuhan, People’s Republic of China; College of Veterinary Medicine, Cornell University

**Keywords:** gut microbiome, *Streptococcus*, 16S rRNA gene sequencing, gastric cancer, metastatic

## Abstract

The gut microbiome plays an indispensable role in the occurrence and progression of various diseases. However, its ability to predict gastric cancer (GC) and liver metastasis (GCLM) has not been fully identified. Fecal samples were collected from 49 GC patients (cancer group [group C]) and 49 healthy people (normal group [group N]) between 4 July 2020 and 9 March 2021. Furthermore, 26 patients with metastatic GC were divided into a liver metastatic group (group L) (*n* = 13) and a non-liver-metastatic group (group M) (*n* = 13). DNA was extracted, and 16S rRNA gene sequencing was performed. SPSS was used for statistical analyses, and all bioinformatics analyses were based on QIIME2. *P* values of <0.05 were considered statistically significant. The microbial richness and diversity in group C were higher than those in group N, and there were significant differences in species compositions between the two groups. Streptococcus, enriched in groups C and L by linear discriminant analysis (LDA) effect size (LEfSe) and further identified by a random forest (RF) model, enhances its potential as a biomarker for GC and GCLM. Functional gene and metabolic pathway analyses showed that d-galacturonate degradation pathway II was of great importance in the occurrence and development of GC. Streptococcus has the potential ability to predict GC and GCLM, which is critical for the early diagnosis of GC and GCLM.

## INTRODUCTION

According to 2020 global cancer statistics from the International Agency for Research on Cancer (IARC), gastric cancer (GC) is the fifth leading cause of new cases (5.6%) and the fourth leading cause of cancer-related deaths (7.7%) worldwide (1), and the 5-year survival rate in China is 27.4% (2). Although the development of diagnostic techniques and alternation of treatment methods have improved the curative effect of on GC the median overall survival (OS) of patients with liver metastasis is only 2 months without systemic chemotherapy (3). Therefore, early detection, diagnosis, and treatment are of great importance. Endoscopy is a noninvasive and highly precise examination for early GC (4), but it requires an interventional oral operation, which is difficult for patients to tolerate, so it is difficult for GC screening and suitable for only a few people.

Gastric carcinogenesis is a multifactorial process that can be affected by host status (age, gender, family history, Eastern Cooperative Oncology Group [ECOG] performance, smoking, drinking, high-salt diet, high-fat diet, and history of surgery, etc.) (5), environmental factors (gut microbiome, gastric acid pH value, and Helicobacter pylori and Epstein-Barr virus [EBV] infections, etc.), and genetic factors. H. pylori infection is the strongest risk factor for GC, especially noncardiac GC, which can directly cause genetic instability by DNA double-stranded breaks, thus directly promoting the occurrence and development of GC (6). Nevertheless, only about 3% of H. pylori infections will eventually develop into GC (7); therefore, it is vital to find an intestinal microorganism that can more accurately predict the occurrence of GC. There are about 10^14^ bacteria and thousands of species in the human digestive tract, distributed mainly in the colon and rectum (8), which makes fecal samples suitable for intestinal microbiome analysis.

We hypothesized that a specific microorganism was involved in promoting GC and liver metastasis (GCLM). For verification, 16S rRNA gene sequencing was used to analyze the content and diversity in fecal samples from GC patients. 16S rRNA is a component of the ribosomal 30S subunit in prokaryotes, which is highly conservative and specific. The 16S rRNA gene is a DNA sequence corresponding to rRNA coding in bacteria, including conserved regions and variable regions. The former reflects the genetic relationship between species, while the latter reflects the differences between species. The V3-V4 variable region of the 16S rRNA gene was sequenced to study microbial diversity (9).

Based on this background, we aimed to find a biomarker of the gastrointestinal microbiota to predict GC and liver metastasis and to explore its role in early diagnosis and treatment.

## RESULTS

### Demographic characteristics of the GC patients.

A total of 49 GC patients between 4 July 2020 and 9 March 2021 were included in this retrospective study. The baseline characteristics of 49 patients are summarized in Table 1. The median age (range) was 62 (52 to 67) years, including 18 (36.73%) females and 31 (63.27%) males. The median body mass index (BMI) (range) was 20.96 (19.31 to 22.50) kg/cm^2^. Ten cases (20.41%) had GC in the gastric antrum, and the rest (79.59%) had GC that was not located in the antrum. The histology of most GC patients (64.44%) showed poor differentiation. Twenty-six (53.06%) patients had metastasis during treatments. The patients were followed up to 1 August 2021, the median duration of progression-free survival (PFS) was 6.07 months (*n* = 33).

**TABLE 1:** Demographic characteristics of GC patients (*n* = 49)

Variable^*a*^	Value
Median age (yrs) (range)	62 (52–67)
No. (%) of patients of gender	
Male	31 (63.27)
Female	18 (36.73)
Median BMI (kg/cm^2^) (range) (*n* = 47)	20.96 (19.31–22.50)
No. (%) of patients with tumor site	
Antrum	10 (20.41)
Nonantrum	39 (79.59)
No. (%) of patients with histology	
Low differentiation	29 (64.44)
Medium differentiation	3 (6.12)
High differentiation	2 (4.08)
Unknown	15 (30.61)
No. (%) of patients with metastasis	
Yes	26 (53.06)
No	23 (46.94)
No. (%) of patients with liver metastasis (*n* = 26)	
Yes	13 (50.00)
No	13 (50.00)
No. (%) of patients with surgery	
Yes	30 (61.22)
No	19 (38.78)
No. (%) of patients with no. of lines of treatment of:	
<3	26 (53.06)
≥3	7 (14.29)
None	16 (32.53)
No. (%) of patients with ECOG score of:	
0	29 (59.18)
1	15 (30.61)
2	5 (10.20)
No. (%) of patients with clinical response	
PD	17 (34.69)
Non-PD	20 (40.82)
Unknown	12 (24.49)
Median NLR (range)	1.91 (1.29–2.63)
Median PLR (range)	147.50 (103.94–210.09)
Median MLR (range)	0.22 (0.18–0.32)
Median CA125 (U/ml) (range) (*n* = 39)	23.30 (11.90–62.10)
Median CA199 (U/ml) (range) (*n* = 48)	7.10 (3.15–19.30)
Median CEA (μg/liter) (range) (*n* = 48)	1.97 (1.26–5.12)
Median CA153 (U/ml) (range) (*n* = 26)	7.45 (5.30–9.90)
Median ALB (g/liter) (range)	38.70 (36.10–41.75)
Median GLB (g/liter) (range)	26.20 (22.55–28.90)
Median LDH (U/liter) (range)	165.00 (146.00–183.00)
Median ALP (U/liter) (range)	85.00 (73.00–118.50)
Median progress-free survival (mos) (range) (*n* = 33)	6.07 (3.32–8.75)

a Abbreviations: NLR, neutrophil-to-lymphocyte ratio; PLR, platelet-to-lymphocyte ratio; MLR, monocyte-to-lymphocyte ratio; CA125, carbohydrate antigen 125; CEA, carcinoembryonic antigen; ALB, albumin; GLB, globulin; LDH, lactate dehydrogenase; ALP, alkaline phosphatase. Range, quartiles (P_25_, P_75_).

### Sequencing data processing.

**FIG 1:**
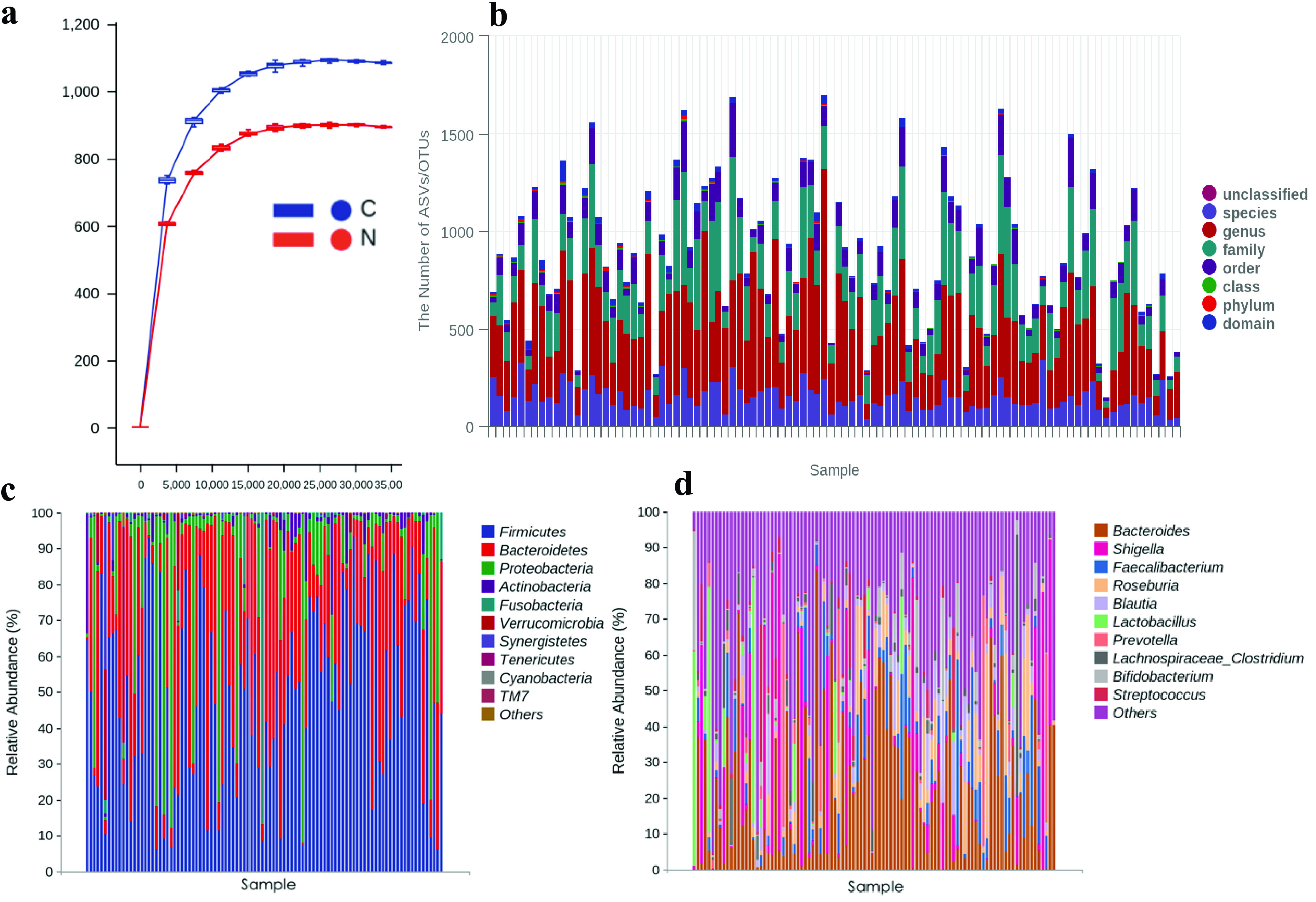
Species compositions and microbial diversity analyses of the C and N groups. (a) Rarefaction curve. (b) Species taxonomy in each sample. (c and d) Microbial composition at the phylum level (c) and the genus level (d). Note that the groups in the abscissa are displayed in the order C1 to C49 and N1 to N49.

We took the numbers of sequences as the abscissa and the corresponding numbers of operational taxonomic units (OTUs) as the ordinate to draw the rarefaction curve, as shown in Fig. 1a. In this study, the end of the curves for the group of GC patients (cancer group [group C]) and the group of healthy individuals (normal group [group N]) tend to be flat, indicating that the amount of sequencing data is reasonable, more data will produce only a small number of new OTUs, and the sequencing depth is enough to cover most bacteria. Among them, the richness of group C samples is higher than that of group N samples.

### 16S rRNA sequencing results. (i) ASV/OTU cluster analysis and species annotation.

A total of 45,643 OTUs were obtained in this study, including 27,250 in group C, 21,740 in group N, and 3,347 in both groups C and N. The average number of OTUs is 894. The amplicon sequence variants (ASVs) ranged in length from 49 to 442 bp, with a total of 2,280,865,555 bp and an average length of 418 bp. On average, 1 domain, 7 phyla, 14 classes, 18 orders, 32 families, 46 genera, and 37 species were identified. A histogram was created to show the microbial composition of samples in groups C and N (Fig. 1b).

### (ii) Differences in bacterial compositions at the phylum and genus levels.

In all samples, 243 phyla and 38,706 genera were annotated, with averages of 3 phyla and 395 genera, respectively. Figure 1c shows the 10 most abundant bacteria at the phylum level, *Firmicutes*, *Bacteroidetes*, *Proteobacteria*, *Actinobacteria*, *Fusobacteria*, *Verrucomicrobia*, *Synergistetes*, *Tenericutes*, *Cyanobacteria*, and TM7, while Fig. 1d shows the 10 most abundant bacteria at the genus level, *Bacteroides*, *Shigella*, *Faecalibacterium*, *Roseburia*, *Blautia*, *Lactobacillus*, *Prevotella*, *Lachnospiraceae_Clostridium*, *Bifidobacterium*, and Streptococcus, which accounted for more than 95% of the total microbiota abundance.

### (iii) Microbial diversity analysis.

**FIG 2:**
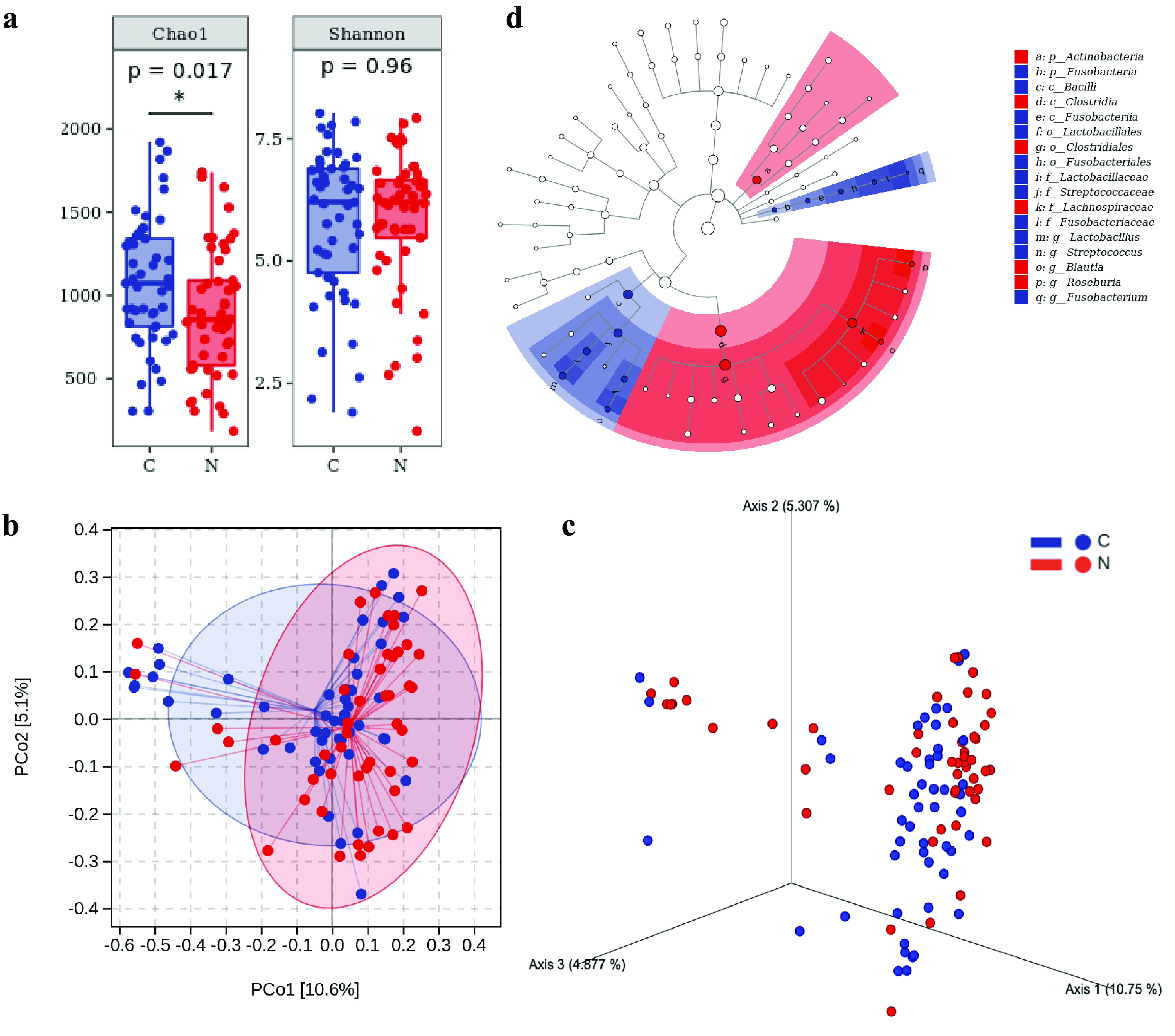
Microbial diversity and LEfSe analyses of the C and N groups. (a) Alpha diversity measurements by species richness and gene counts. (b and c) Beta diversity measurements by PCoA (b) and 3D-PCoA (c). (d) Taxonomic branch diagram of significant microbial species (LDA threshold of 4).

As for alpha diversity, the species richness of group C was higher than that of group N (*P *= 0.017), but there were no significant differences in species diversity between groups C and N (*P* = 0.96) (Fig. 2a). According to principal-coordinate analysis (PCoA), PCo1 is 10.75%, PCo2 is 5.31%, PCo3 is 4.88%, and the three coordinate axes represent 20.94% of the C and N groups. The beta diversity difference between the C and N groups was large (*R*^2^ = 0.0285; *P* = 0.001), indicating that there were composition differences between the two groups. Figure 2b and c represent the PCoAs and three-dimensional PCoAs (3D-PCoAs) for the C and N groups.

We set the linear discriminant analysis (LDA) threshold to 4 and conducted LDA effect size (LEfSe) to explore the microbial species with significant differences between groups C and N. A total of 17 enriched species were identified (Fig. 2d). Among them, 11 species are enriched in group C, ranking from high to low according to LDA values as follows: *c_Bacilli*, *o_Lactobacillales*, *f_Lactobacillaceae*, *g_Lactobacillus*, *f_Fusobacteriaceae*, *o_Fusobacteriales*, *p_Fusobacteria*, *c_Fusobacteriia*, *f_Streptococcaceae*, *g_*Streptococcus, and *g_Fusobacterium.* In group N, the six species sorted by LDA values from high to low were *f_Lachnospiraceae*, *c_Clostridia*, *o_Clostridiales*, *g_Roseburia*, *g_Blautia*, and *p_Actinobacteria.*

### (iv) Random forest classification model.

Table 2 and Fig. S1a in the supplemental material show the top five intestinal microorganisms at the genus level with the ability to predict GC using the random forest (RF) model: (i) ASV-126225, *Ochrobactrum* (area under the curve [AUC] = 0.139; *P < *0.001); (ii) ASV-92932, *Aquabacterium* (AUC = 0.245; *P* < 0.001); (iii) ASV-61493, Streptococcus 1 (AUC = 0.772; *P* < 0.001); (iv) ASV-63689, Streptococcus 2 (AUC = 0.842; *P* < 0.001); and (v) ASV-84276, *Lachnospiraceae* (AUC = 0.244; *P* < 0.001). Streptococcus was represented by ASV-61493 and ASV-63689, yielding AUCs of >0.7 (Fig. S1b), indicating that the model had a good effect in predicting GC, which was also identified as a GC-related microbial species by LEfSe, enhancing the potential of being a biomarker for GC diagnosis.

**TABLE 2:** Random-forest model to predict biomarkers for GC diagnosis

Rank	ASV	Bacterial taxon	AUC	SE for AUC	*P* value	Group enriched by LEfSe
1	ASV-126225	*Ochrobactrum*	0.139	0.039	<0.001	
2	ASV-92932	*Aquabacterium*	0.245	0.050	<0.001	
3	ASV-61493	Streptococcus 1	0.772	0.049	<0.001	C
4	ASV-63689	Streptococcus 2	0.842	0.042	<0.001	C
5	ASV-84276	*Lachnospiraceae*	0.244	0.050	<0.001	N

10.1128/mBio.02994-21.1 FIG S1 Random forest model analysis and survival analysis. (a) Species composition heat map for the RF model. (b) RF model tested by ROC curve analysis. (c to e) Survival analysis (*n* = 33) curve with low and high expression levels of Streptococcus 1 (c), Streptococcus 2 (d), and *Lachnospiraceae* (e). Download FIG S1, PDF file, 0.3 MB. Copyright © 2021 Yu et al. 2021 Yu et al. https://creativecommons.org/licenses/by/4.0/ This content is distributed under the terms of the Creative Commons Attribution 4.0 International license.

### (v) Survival analysis based on Streptococcus 1/2 and *Lachnospiraceae*.

The survival of GC patients (*n* = 33) with the microorganisms Streptococcus 1 (ASV-61493), Streptococcus 2 (ASV-63689), and *Lachnospiraceae* (ASV-84276) with predictive function in the RF model was analyzed. The cutoff value was calculated by receiver operating characteristic (ROC) curves, the values of which were 1.5, 20.5, and 6.5 for the three microorganisms, respectively, and the microorganisms were divided into high- and low-expression groups. It was found that the PFS durations were long in the Streptococcus 1 low-expression group (*P* = 0.898), short in the Streptococcus 2 low-expression group (*P *= 0.726), and long in the *Lachnospiraceae* low-expression group (*P* = 0.986) (for details, see Fig. S1c to e).

### KEGG function analysis and KO metabolic pathway annotations for the C and N groups.

The 16S rRNA sequencing results were analyzed by Kyoto Encyclopedia of Genes and Genomes (KEGG) and KEGG Orthology (KO) analyses, and 7 function classifications and 60 metabolic pathways were annotated for the C and N groups. Table S1 shows the average relative abundances of functional pathway classifications from high to low, followed by biosynthesis, degradation/utilization/assimilation, glycan pathways, the generation of precursor metabolites and energy, metabolic clusters, macromolecule modification, and detoxification. The top nine metabolic pathways with significance are shown in Table S2. d-Galacturonate degradation pathway II plays the most vital role in the occurrence and development of GC.

10.1128/mBio.02994-21.2 TABLE S1 KEGG functional pathway analysis of the C and N groups. Download Table S1, DOCX file, 0.02 MB. Copyright © 2021 Yu et al. 2021 Yu et al. https://creativecommons.org/licenses/by/4.0/ This content is distributed under the terms of the Creative Commons Attribution 4.0 International license.

10.1128/mBio.02994-21.3 TABLE S2 KEGG Orthology metabolic pathway annotations for the C and N groups. Download Table S2, DOCX file, 0.01 MB. Copyright © 2021 Yu et al. 2021 Yu et al. https://creativecommons.org/licenses/by/4.0/ This content is distributed under the terms of the Creative Commons Attribution 4.0 International license.

### Gut microbiota effects on GC patients with liver metastasis.

**FIG 3:**
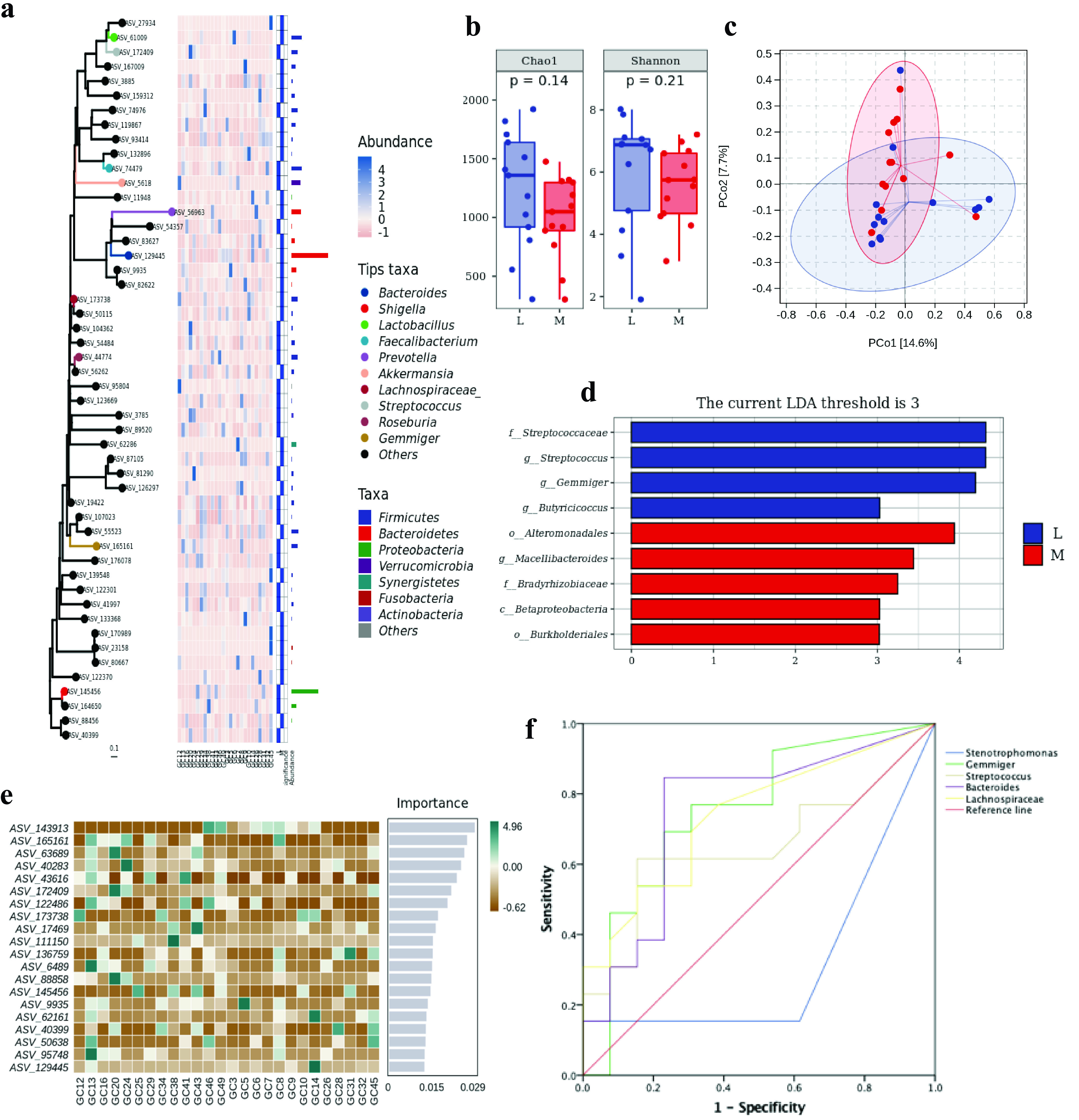
Microbial diversity and random forest model analysis between the L and M groups. (a) Phylogenetic tree plot of microbial composition at the phylum and genus levels. (b) Alpha diversity measurements by species richness and gene counts. (c) Beta diversity measurements by PCoA. (d) LDA histogram of significant microbial species (LDA threshold of 3). (e) Species composition heat map in the RF model. (f) RF model tested by ROC curve analysis.

To further explore the effect of fecal bacteria on GC patients with liver metastasis, we divided them into a liver metastatic group (group L) (*n* = 13) and a non-liver-metastatic group (group M) (*n* = 13). There was no significant difference in clinicopathological features between the two groups (Table S4). According to the phylogenetic tree plot of the microbial compositions of groups L and M, the 10 most abundant bacteria at the genus level were *Bacteroides*, *Shigella*, *Lactobacillus*, *Faecalibacterium*, *Prevotella*, *Akkermansia*, *Lachnospiraceae*, *Roseburia*, Streptococcus, *Gemmiger*, and others, which were very different in the C and N groups (Fig. 3a). For microbial diversity, species richness (*P* = 0.14) and diversity (*P* = 0.21) were higher in the L group (Fig. 3b), and the beta diversity difference between the L and M groups was large (*R*^2^ = 0.0374; *P* = 0.559) (Fig. 3c). With the LDA threshold set at 3, LEfSe (Fig. 3d) showed that there were 4 species enriched in the L group: *f_Streptococcaceae*, *g_*Streptococcus, *g_Gemmiger*, and *g_Butyricicoccus*. Five species were enriched in the M group: *o_Alteromonadales*, *g_Macellibacteroides*, *f_Bradyrhizobiaceae*, *c_Betaproteobacteria*, and *o_Burkholderiales*. Figure 3e and Table S2 show the top five fecal bacteria that can predict GCLM by the RF model: (i) ASV-143913, *Stenotrophomonas* (AUC = 0.317; *P *= 0.112); (ii) ASV-165161, *Gemmiger* (AUC = 0.751; *P *= 0.029); (iii) ASV-63689, Streptococcus (AUC = 0.651; *P *= 0.191); (iv) ASV-40283, *Bacteroides* (AUC = 0.751; *P *= 0.029); and (v) ASV-43616, *Lachnospiraceae* (AUC = 0.740; *P *= 0.038). LEfSe enriched *Gemmiger* and Streptococcus in the L group, yielding AUCs of >0.6 (Fig. 3f), showing great potential in predicting GCLM. In our previous study, Streptococcus was also a possible biomarker to distinguish GC patients from healthy people (Table 2).

10.1128/mBio.02994-21.4 TABLE S3 Random forest classification prediction model. Download Table S3, DOCX file, 0.01 MB. Copyright © 2021 Yu et al. 2021 Yu et al. https://creativecommons.org/licenses/by/4.0/ This content is distributed under the terms of the Creative Commons Attribution 4.0 International license.

10.1128/mBio.02994-21.5 TABLE S4 Demographic characteristics of GCLM patients (*n* = 26). Download Table S4, DOCX file, 0.02 MB. Copyright © 2021 Yu et al. 2021 Yu et al. https://creativecommons.org/licenses/by/4.0/ This content is distributed under the terms of the Creative Commons Attribution 4.0 International license.

## DISCUSSION

It was once thought that the stomach was a sterile environment. Now, after the discovery of H. pylori, the stomach is considered to be a complex ecosystem containing a variety of gastrointestinal microbes (10). Moreover, several studies have reported that microorganisms may affect the balance and pathogenesis of H. pylori (11, 12), which plays a vital part in GC. Fecal samples are easy to obtain and can represent the vast majority of the microbial environment in the stomach (13). Therefore, in order to find microorganisms that may predict GC and GCLM, we retrospectively collected fecal samples from 49 GC patients (C group) and 49 healthy people (N group), and 16S rRNA gene sequencing was used to further explore the value of early diagnosis and treatment to improve the prognosis of these patients.

Our study found that the most abundant microbes in these two groups were *Bacteroides*, *Shigella*, *Faecalibacterium*, *Roseburia*, *Blautia*, *Lactobacillus*, *Prevotella*, *Lachnospiraceae_Clostridium*, *Bifidobacterium*, and Streptococcus at the genus level. Alpha diversity analysis showed that the richness in group C was significantly higher, and there were significant differences in beta diversity analyses. Additionally, LEfSe was used to find key taxa in the C and N groups. As a result, 17 species were enriched, of which 11 (64.71%) species were in group C and 7 were in group N. *Lactobacillus*, *Fusobacterium*, and Streptococcus were enriched in group C at the genus level, indicating that they might be associated with GC. As a probiotic, *Lactobacillus* can increase the production of lactate, *N*-nitroso compounds, and reactive oxygen species (ROS) as well as the epithelial-mesenchymal transition (EMT), immune tolerance, and colonization by other carcinogens (14), all of which will lead to tumor development, recurrence, metastasis, and poor prognoses (15). Yu et al. (16) found that *Fusobacterium* can target Toll-like receptor (TLR) immune signals and specific microRNAs to activate the autophagy pathway and further promote chemotherapy resistance of cancer, especially colorectal cancer (CRC), which was a conclusion similar to that of Bullman et al. (17). However, so far, the pathogenic mechanism of Streptococcus is not clear; elevated levels of interleukin-8 (IL-8), cyclooxygenase 2 (COX2), and cell proliferation might contribute to its carcinogenesis (18).

Furthermore, when the RF model was constructed, Streptococcus 1 and 2 in group C and *Lachnospiraceae* in group N were the top five microorganisms classified by LEfSe, yielding AUCs of 0.772, 0.842, and 0.244, respectively, which demonstrated that Streptococcus had the ability to predict GC. In addition, Streptococcus was also identified as a microbe that is able to predict GCLM by comparing the liver metastasis group (group L) with the non-liver-metastasis group (group M). However, by survival analysis, Streptococcus 1 and 2 and the other three species were not prognostic factors for GC. Moreover, KEGG analysis showed that d-galacturonate degradation pathway II plays the most vital role in GC occurrence and development.

Streptococcus is a common pyogenic Gram-positive coccus, which widely exists in the human gastrointestinal tract and nasopharynx, mainly causing pyogenic inflammation, hypersensitivity diseases, and so on. Our study found that Streptococcus can predict the occurrence of GC and GCLM. Therefore, we believe that direct smear microscopy of fecal/pharyngeal swabs is a simple and economical method to determine the possibility of GC, but the result needs to be differentiated from inflammatory diseases by comprehensive observation of hematological inflammatory indices and the basic status of patients, etc. Intestinal microorganisms can predict the occurrence of GC and GCLM, suggesting that we can use the existence of microbes as a screening method to prevent the occurrence of gastric cancer, which can suggest further endoscopy, imaging, pathology, and other examinations to detect diseases early, treat patients in advance, and improve the prognosis of patients. However, there are some limitations to our study: (i) this study is limited to a single center with a small sample size, which needs to be externally verified by large sample sizes and multicenter trials; (ii) some patients had undergone radical gastrectomy/exploratory laparotomy or bowel preparation before gastroenteroscopy, which may affect intestinal microorganisms; and (iii) the region, dietary structure, customs, and other differences of the included patients may have a certain impact on the results. Despite the above-mentioned defects, this study can still explain the role of Streptococcus in GC and GCLM to a great extent.

### Conclusion.

We demonstrated significant differences in intestinal microbiome compositions between GC patients and healthy people and found that Streptococcus is a potential biomarker for the early prediction of GC and GCLM, which may play a key role in the early diagnosis of diseases.

## MATERIALS AND METHODS

### Patients.

We retrospectively analyzed 49 untreated GC patients (cancer group [group C]) and 49 matched healthy people (normal group [group N]) between 4 July 2020 and 9 March 2021. Furthermore, 26 patients with metastatic GC were divided into a liver metastatic group (group L) (*n* = 13) and a non-liver-metastatic group (group M) (*n* = 13). All cases were pathologically diagnosed by biopsy through gastroscopy or surgery at the cancer center of Union Hospital, Tongji Medical College, Huazhong University of Science and Technology. The inclusion criteria were as follows: (i) the patient had not used probiotics, antibiotics, nonsteroidal anti-inflammatory drugs (NSAIDs), immunosuppressants, chemotherapy drugs, and other drugs affecting the intestinal microbiota 1 month before the collection of fecal samples; (ii) no invasive operation was performed within 7 days before fecal sample collection; (iii) the patient had no history of gastrointestinal (liver, gallbladder, pancreas, and spleen) diseases, such as inflammatory bowel disease (IBD), irritable bowel syndrome (IBS), and chronic constipation, and surgical history (excluding radical gastrectomy and exploratory laparotomy related to this treatment); (iv) the patient had no history of alcoholism, smoking, and drug abuse; and (v) the patient voluntarily participated in the study and signed the informed consent form. Patients who did not meet the inclusion criteria were excluded.

For all GC patients, baseline clinical-pathological characteristics, including age, gender, tumor sites, histology, history of metastasis, history of surgery, lines of treatment, Eastern Cooperative Oncology Group (ECOG) performance, clinical response, and baseline blood counts, were available for review. All patients were monitored regularly until death or the study data cutoff (1 August 2021). According to Response Evaluation Criteria in Solid Tumors (RECIST 1.1), the clinical response was divided into progressive disease (PD) and nonprogressive disease (non-PD) (19) in metastasis GC patients by imaging examination, while among patients with nonmetastatic GC, those without progression were considered to be non-PD patients. Baseline blood counts were defined as results obtained within 7 days before fecal samples were collected. This retrospective study was approved by the Ethics Committee of Union Hospital, Tongji Medical College, Huazhong University of Science and Technology (approval no. 2014-041).

### Sample collection.

Fecal samples were collected by a natural defecation method. Urine was drained before defecation to avoid pollution. A sampling spoon was used to put 2 g of feces into a sterile fecal sample collection tube, which was then placed into an icebox to create a low-temperature environment to prevent bacterial growth. Fecal samples were stored in a −80°C refrigerator within 2 h. If fecal samples were not easily available, a rectal swab method was used to collect fecal samples.

### DNA extraction.

Total microbiome DNA was isolated from the fecal samples of 49 GC patients and 49 healthy controls using the Omega Mag-Bind soil DNA kit (Omega Bio-Tek, Norcross, GA, USA). The quality of the extracted DNA was determined by 0.8% agarose gel electrophoresis. The DNA fragments were subjected to paired-end (PE) sequencing on an Illumina platform, and the original sequencing data were saved in FASTQ format.

### 16S rRNA metagenomic sequencing.

The Illumina sequencing method was used to amplify the V3-V4 variable region of 16S rRNA of the bacterial genome by PCR. The forward and reverse primer sequences were 5′-ACTCCTACGGGAGGCAGCA-3′ and 5′-GGACTACHVGGGTWTCTAAT-3′, respectively. The high-throughput sequencing library was constructed by using the Illumina TruSeq Nano DNA LT library prep kit (Illumina, San Diego, CA, USA). The dada2 method of Quantitative Insights into Microbial Ecology2 (QIIME2) software (v2019.4) (20) was used to optimize the original sequences, mainly including input, filtering, denoising, merging, chimerism, and singleton. The deduplicated sequences were amplicon sequence variants (ASVs) with approximately 100% identity, and the UPARSE-OUT algorithm in VSERACH software (v2.13.4_linux_x86_64) was utilized to select paired sequences at a 97% identity match to operational taxonomic units (OTUs). We used QIIME2 software to allocate the representative sequences taxonomically by searching the Greengenes database (http://greengenes.secondgenome.com/) (21) with default parameters, and the pretrained naive Bayes classifier (https://github.com/QIIME2/q2-feature-classifier) (22) was used for species annotation.

### Statistical and bioinformatic analyses.

SPSS version 22.0 (SPSS Inc., Chicago, IL, USA) was used for statistical analysis. GraphPad Prism 8.0 (GraphPad Software Inc., San Diego, CA, USA) was used to draw charts. The baseline parameters of the included patients were displayed as medians (quartiles) [*M* (*P*_25_, *P*_75_)]. The patient characteristics were compared by a chi-squared test and Student’s *t* test. Progression-free survival (PFS) was determined from the date of pathology diagnosed as GC to the date of progression proven by radiographic assessment, obvious clinical manifestations, death from any cause, or the last follow-up (censored). Survival analyses were conducted using the Kaplan-Meier method and the Cox proportional-hazards model. All tests performed were two sided, and *P* values of <0.05 were considered statistically significant.

According to the ASV/OTU abundance matrix, R software was used to draw rarefaction curves and species accumulation curves. Microbial alpha diversity analysis was carried out in QIIME2 software, and community richness and diversity were determined by Chao1 and Shannon indices (23), respectively. A Kruskal-Wallis test was used to verify significance. Beta diversity was assessed according to the differences in microbial community structures among groups using principal-coordinate analysis (PCoA) based on Bray-Curtis distance (24), and the differences between groups were analyzed by permutational multivariate analysis of variance (PERMANOVA). Linear discriminant analysis (LDA) effect size (LEfSe) (25) was applied to analyze the differences in microbial communities, aiming to find biomarkers. By default, the LDA threshold is set to 4. To explore the potential GC diagnostic ability of intestinal flora, we constructed a random forest (RF) model based on the 100 ASVs with the most significant differentiation. A randomly selected 50% of the data were used as the training data set to train and build the model, the other half of the data were used as the validation data set to verify the accuracy of the model, and the fitting model was verified by receiver operating characteristic (ROC) and area under the curve (AUC) analyses (26). We used PICRUSt2.0 from the Kyoto Encyclopedia of Genes and Genomes (KEGG) database and the MetaCyc database to perform KEGG Orthology (KO) analysis and predict microbial metabolism function.
