# Supplementary figures and images for: Fecal Streptococcus Alteration Is Associated with Gastric Cancer Occurrence and Liver Metastasis
Source: mBio. 2021 Dec 7;12(6):e02994-21. doi: 10.1128/mBio.02994-21 (PMC8649758; doi:10.1128/mBio.02994-21)

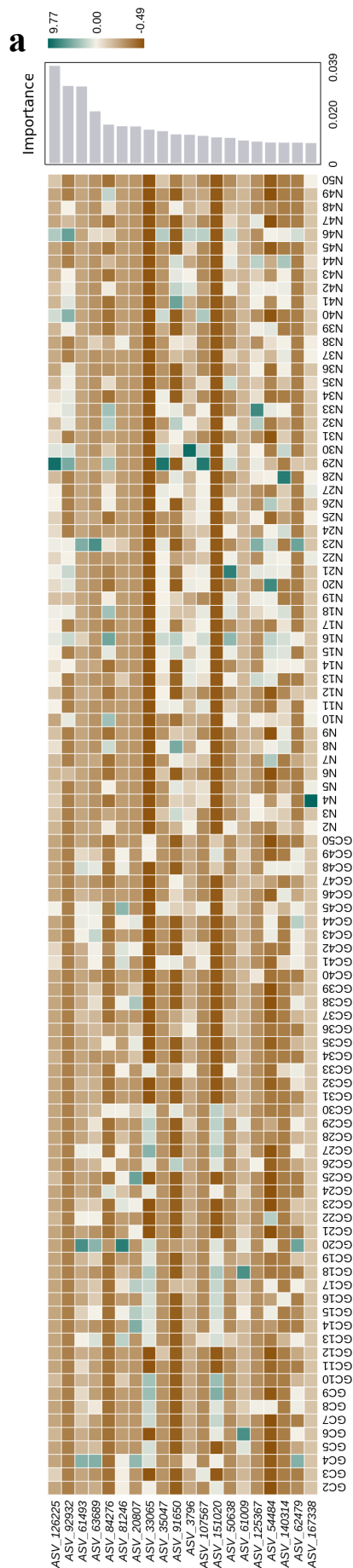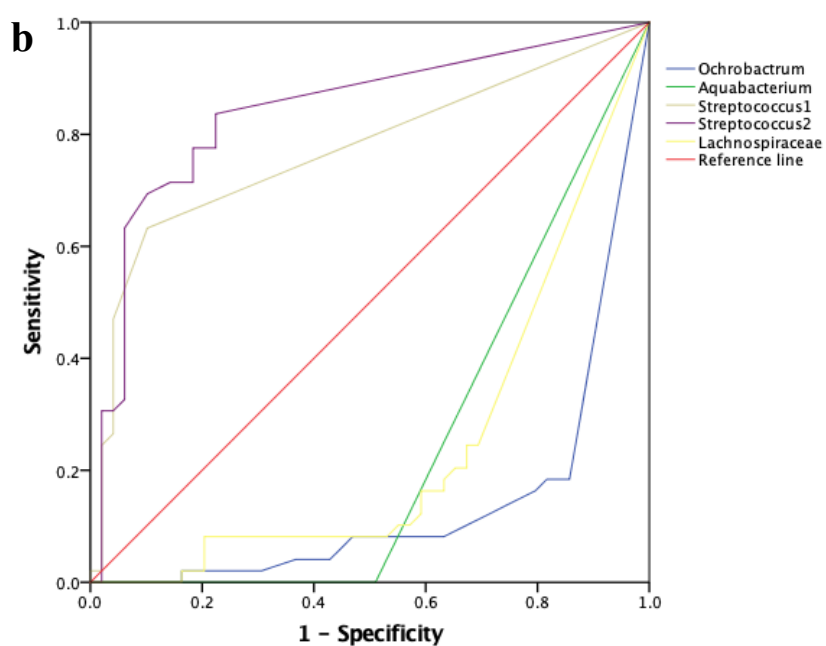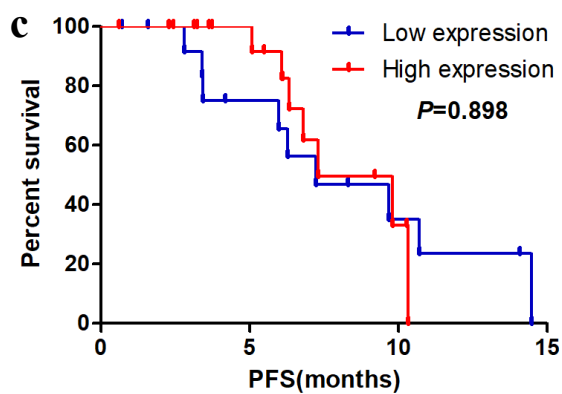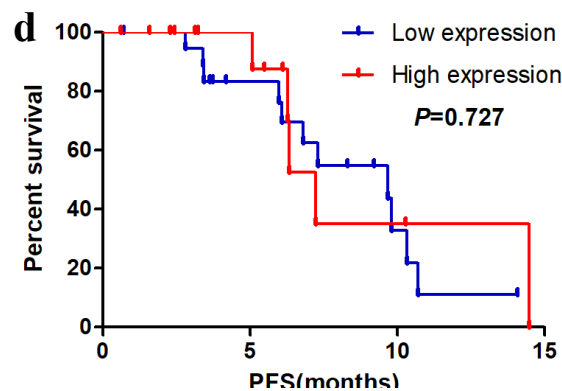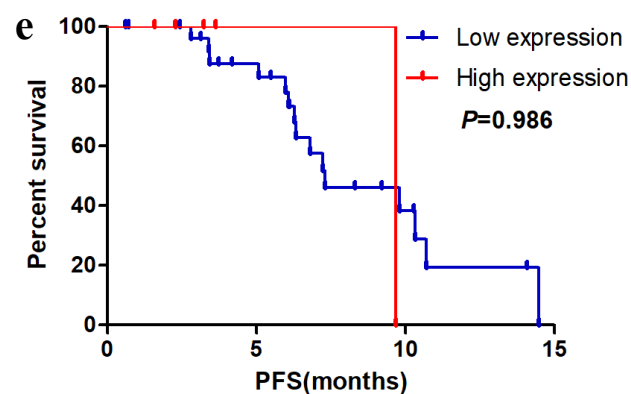

Supplement: FIG S1 [file mbio.02994-21-sf001.pdf]
