# Supplementary material for: Fecal Streptococcus Alteration Is Associated with Gastric Cancer Occurrence and Liver Metastasis
Source: mBio. 2021 Dec 7;12(6):e02994-21. doi: 10.1128/mBio.02994-21 (PMC8649758; doi:10.1128/mBio.02994-21)
Supplement: TABLE S1 [file mbio.02994-21-st001.docx]

**Supplementary Table 1 KEGG functional pathway analysis in C and N group**

| **Function**  **classifications** | **Metabolic pathways** | **Relative**  **abundance** | **Function**  **classifications** | **Metabolic pathways** | **Relative**  **abundance** |
| --- | --- | --- | --- | --- | --- |
| Biosynthesis  (n=12)  (average=12713.72) | Amine and Polyamine Biosynthesis | 904.04 | Generation of Precursor Metabolite and Energy  (n=16)  (average=1522.09) | 1,5-anhydrofructose degradation | 0.43 |
|  | Amino Acid Biosynthesis | 36572.99 |  | Electron Transfer | 79.45 |
|  | Aminoacyl-tRNA Charging | 1433.52 |  | ethylmalonyl-CoA pathway | 1.04 |
|  | Aromatic Compound Biosynthesis | 2913.17 |  | Fermentation | 10195.53 |
|  | Carbohydrate Biosynthesis | 12598.63 |  | formaldehyde oxidation I | 139.83 |
|  | Cell Structure Biosynthesis | 9350.34 |  | Glycolysis | 4325.78 |
|  | Cofactor, Prosthetic Group, Electron Carrier, and Vitamin Biosynthesis | 30282.19 |  | glyoxylate cycle | 178.33 |
|  | Fatty Acid and Lipid Biosynthesis | 18441.68 |  | isopropanol biosynthesis | 22.6 |
|  | Metabolic Regulator Biosynthesis | 215.52 |  | methyl ketone biosynthesis | 2.09 |
|  | Nucleoside and Nucleotide Biosynthesis | 33387.04 |  | methylaspartate cycle | 3.04 |
|  | Other Biosynthesis | 692.15 |  | Pentose Phosphate Pathways | 3025.83 |
|  | Secondary Metabolite Biosynthesis | 5773.41 |  | Photosynthesis | 1765.7 |
| Degradation/  Utilization/  Assimilation  (n=16)  (average=2478.92) | Alcohol Degradation | 332.33 |  | Respiration | 347.89 |
|  | Aldehyde Degradation | 93.47 |  | superpathway of glycolysis and Entner-Doudoroff | 530.48 |
|  | Amine and Polyamine Degradation | 1025.57 |  | superpathway of glycolysis, pyruvate dehydrogenase, TCA, and glyoxylate bypass | 249.33 |
|  | Amino Acid Degradation | 1122.44 |  | TCA cycle | 3486.07 |
|  | Aromatic Compound Degradation | 375.72 | Metabolic Clusters  (n=10)  (average=702.60) | L-glutamate and L-glutamine biosynthesis | 1026.01 |
|  | C1 Compound Utilization and Assimilation | 3661.21 |  | O-antigen building blocks biosynthesis (E. coli) | 1056.14 |
|  | Carbohydrate Degradation | 9803.77 |  | phospholipases | 0.05 |
|  | Carboxylate Degradation | 6665.92 |  | pyrimidine deoxyribonucleotide phosphorylation | 725.06 |
|  | Chlorinated Compound Degradation | 0.12 |  | pyrimidine deoxyribonucleotides biosynthesis from CTP | 174.1 |
|  | Cofactor, Prosthetic Group, Electron Carrier Degradation | 0.02 |  | pyrimidine deoxyribonucleotides de novo biosynthesis I | 706.28 |
|  | Degradation/Utilization/Assimilation - Other | 49.56 |  | pyrimidine deoxyribonucleotides de novo biosynthesis III | 441.53 |
|  | Fatty Acid and Lipid Degradation | 229.54 |  | pyrimidine deoxyribonucleotides de novo biosynthesis IV | 143.7 |
|  | Inorganic Nutrient Metabolism | 1546.73 |  | superpathway of L-aspartate and L-asparagine biosynthesis | 1319.6 |
|  | Nucleoside and Nucleotide Degradation | 5855.38 |  | tRNA charging | 1433.52 |
|  | Polymeric Compound Degradation | 4208.01 | Macromolecule Modification  (n=2)  (average=534.98) | Nucleic Acid Processing | 1069.94 |
|  | Secondary Metabolite Degradation | 4692.93 |  | Protein Modification | 0.02 |
| Glycan Pathways  (n=2)  (average=1626.40) | Glycan Biosynthesis | 1549.65 | Detoxification  (n=2)  (average=358.25) | Antibiotic Resistance | 716 |
|  | Glycan Degradation | 1703.15 |  | methanol oxidation to carbon dioxide | 0.49 |
