# Supplementary material for: Fecal Streptococcus Alteration Is Associated with Gastric Cancer Occurrence and Liver Metastasis
Source: mBio. 2021 Dec 7;12(6):e02994-21. doi: 10.1128/mBio.02994-21 (PMC8649758; doi:10.1128/mBio.02994-21)
Supplement: TABLE S2 [file mbio.02994-21-st002.docx]

**Supplementary Table 2 KEGG Orthology metabolic pathway annotation in C and N group**

| **Pathway** | **LogFC** | **SE** | ***P* values** |
| --- | --- | --- | --- |
| D-galacturonate degradation II | -0.1934 | 0.03616 | <0.0001 |
| Super pathway of salicylate degradation | -1.388 | 0.3393 | 0.0035 |
| catechol degradation III (ortho-cleavage pathway) | -1.426 | 0.3418 | 0.0035 |
| aromatic compounds degradation via beta-ketoadipate | -1.426 | 0.3418 | 0.0035 |
| 4-methylcatechol degradation (ortho cleavage) | -1.429 | 0.3482 | 0.0035 |
| catechol degradation to beta-ketoadipate | -1.405 | 0.3569 | 0.0056 |
| methanol oxidation to carbon dioxide | -1.378 | 0.36 | 0.0074 |
| nicotinate degradation I | -2.505 | 0.7206 | 0.0259 |
| toluene degradation III (aerobic) (via p-cresol) | -1.032 | 0.3108 | 0.0406 |
