# Supplementary material for: Fecal Streptococcus Alteration Is Associated with Gastric Cancer Occurrence and Liver Metastasis
Source: mBio. 2021 Dec 7;12(6):e02994-21. doi: 10.1128/mBio.02994-21 (PMC8649758; doi:10.1128/mBio.02994-21)
Supplement: TABLE S3 [file mbio.02994-21-st003.docx]

**Supplementary Table 3 Random Forest classification prediction model**

| Order | ASV | Bacteria | AUC | SE | *P* | Enriched group by LEfSe |
| --- | --- | --- | --- | --- | --- | --- |
| 1 | ASV-143913 | *Stenotrophomonas* | 0.317 | 0.113 | 0.112 | / |
| 2 | ASV-165161 | *Gemmiger* | 0.751 | 0.100 | 0.029 | L |
| 3 | ASV-63689 | *Streptococcus* | 0.651 | 0.113 | 0.191 | L |
| 4 | ASV-40283 | *Bacteroides* | 0.751 | 0.102 | 0.029 | / |
| 5 | ASV-43616 | *Lachnospiraceae* | 0.740 | 0.099 | 0.038 | / |
