# Supplementary material for: Fecal Streptococcus Alteration Is Associated with Gastric Cancer Occurrence and Liver Metastasis
Source: mBio. 2021 Dec 7;12(6):e02994-21. doi: 10.1128/mBio.02994-21 (PMC8649758; doi:10.1128/mBio.02994-21)
Supplement: TABLE S4 [file mbio.02994-21-st004.docx]

**Supplementary Table 4 Demographic characteristics of GCLM patients (N=26)**

| **Variable** | **Total**  **(n=26)(%)** | **Liver metastasis**  **(n=13)(%)** | **Non-liver metastasis**  **(n=13)(%)** | **χ^2^/t** | ***P* value** |
| --- | --- | --- | --- | --- | --- |
| Age (y) | 63 (51-67) | 63 (52-67) | 62 (49-69) | 0.408 | 0.687 |
| Gender |  |  |  |  |  |
| Male | 19 (%) | 10 (%) | 9 (%) | 0.195 | 0.658 |
| Female | 7 (%) | 3 (%) | 4 (%) |  |  |
| Tumor sites |  |  |  |  |  |
| Antrum | 7 (%) | 4 (%) | 3 (%) | 0.195 | 0.658 |
| Non-antrum | 19 (%) | 9 (%) | 10 (%) |  |  |
| Histology |  |  |  |  |  |
| Low differentiation | 15 (%) | 9 (%) | 6 (%) | 1.418 | 0.234 |
| Medium differentiation | 0 (0.00%) | 0 (0.00%) | 0 (0.00%) |  |  |
| High differentiation | 0 (0.00%) | 0 (0.00%) | 0 (0.00%) |  |  |
| Unknown | 11 (%) | 4 (%) | 7 (%) |  |  |
| Whether surgery |  |  |  |  |  |
| Yes | 11 (%) | 6 (%) | 5 (%) | 0.158 | 0.691 |
| No | 15 (%) | 7 (%) | 8 (%) |  |  |
| Lines of treatment |  |  |  |  |  |
| <3 | 14 (%) | 7 (%) | 7 (%) | 0.000 | 1.000 |
| ≥3 | 4 (%) | 2 (%) | 2 (%) |  |  |
| None | 8 (%) | 4 (%) | 4 (%) |  |  |
| ECOG |  |  |  |  |  |
| 0 | 14 (%) | 8 (%) | 6 (%) | 0.730 | 0.694 |
| 1 | 9 (%) | 4 (%) | 5 (%) |  |  |
| 2 | 3 (%) | 1 (%) | 2 (%) |  |  |
| Clinical response |  |  |  |  |  |
| PD | 6 (%) | 7 (%) | 6 (%) | 0.220 | 0.896 |
| Non-PD | 13 (%) | 3 (%) | 3 (%) |  |  |
| Unknown | 7 (%) | 3 (%) | 4 (%) |  |  |
| NLR | 2.52 (1.60-3.96) | 2.61 (1.66-4.31) | 2.04 (1.58-3.97) | 0.035 | 0.972 |
| PLR | 170.73 (110.27-282.33) | 158.72 (114.17-247.61) | 184.25 (98.94-331.69) | -0.698 | 0.492 |
| MLR | 0.31 (0.21-0.38) | 0.27 (0.22-0.39) | 0.31 (0.19-0.35) | 0.540 | 0.594 |
| CA125 (U/ml) (n=21) | 31.10 (14.45-83.35) | 21.05 (7.78-40.08) | 80.50 (26.60-112.30) | -1.762 | 0.094 |
| CA199 (U/ml) (n=25) | 10.60 (3.40-108.20) | 9.30 (2.85-253.53) | 15.20 (3.75-108.20) | 0.590 | 0.561 |
| CEA (ug/L) (n=25) | 3.09 (1.63-25.29) | 6.63 (1.86-217.83) | 2.03 (1.30-13.45) | 1.205 | 0.240 |
| CA153 (U/ml) (n=17) | 7.50 (5.45-11.55) | 11.90 (6.90-13.70) | 6.80 (5.03-8.80) | 2.014 | 0.062 |
| ALB (g/L) | 37.10 (35.45-40.18) | 37.00 (36.10-38.15) | 37.20 (32.75-42.50) | -0.540 | 0.594 |
| GLB (g/L) | 26.00 (21.68-29.30) | 25.80 (21.80-30.15) | 26.40 (21.45-28.90) | 0.511 | 0.614 |
| LDH (U/L) | 171.50 (144.75-229.75) | 148.00 (131.50-383.50) | 177.00 (156.00-204.00) | 1.465 | 0.156 |
| ALP (U/L) | 78.00 (73.00-116.00) | 98.00 (75.00-225.00) | 73.00 (61.00-101.50) | 1.857 | 0.076 |
| Progress-free survival (m) (n=20) | 6.30 (3.38-9.70) | 6.27 (2.95-8.97) | 6.80 (3.33-9.74) | 0.151 | 0.882 |
